# Predicting meniscal tear stability across knee-joint flexion using finite-element analysis

**DOI:** 10.1007/s00167-018-5090-4

**Published:** 2018-08-10

**Authors:** Angela E. Kedgley, Teng-Hui Saw, Neil A. Segal, Ulrich N. Hansen, Anthony M. J. Bull, Spyros D. Masouros

**Affiliations:** 1 Department of Bioengineering, Imperial College London, Royal School of Mines Building, South Kensington Campus, London, SW7 2AZ UK; 2 Department of Rehabilitation Medicine, University of Kansas Medical Center, 3901 Rainbow Boulevard, Kansas City, KS 66160 USA; 3 Department of Mechanical Engineering, Imperial College London, City and Guilds Building, South Kensington Campus, London, SW7 2AZ UK

**Keywords:** Meniscus, Meniscal tear, Repair, Reconstruction, Surgery, Knee, Biomechanics, Finite element analysis, Arthroscopy

## Abstract

**Purpose:**

To analyse the stress distribution through longitudinal and radial meniscal tears in three tear locations in weight-bearing conditions and use it to ascertain the impact of tear location and type on the potential for healing of meniscal tears.

**Methods:**

Subject-specific finite-element models of a healthy knee under static loading at 0°, 20°, and 30° knee flexion were developed from unloaded magnetic resonance images and weight-bearing, contrast-enhanced computed tomography images. Simulations were then run after introducing tears into the anterior, posterior, and midsections of the menisci.

**Results:**

Absolute differences between the displacements of anterior and posterior segments modelled in the intact state and those quantified from in vivo weight-bearing images were less than 0.5 mm. There were tear-location-dependent differences between hoop stress distributions along the inner and outer surfaces of longitudinal tears; the longitudinal tear surfaces were compressed together to the greatest degree in the lateral meniscus and were most consistently in compression on the midsections of both menisci. Radial tears resulted in an increase in stress at the tear apex and in a consistent small compression of the tear surfaces throughout the flexion range when in the posterior segment of the lateral meniscus.

**Conclusions:**

Both the type of meniscal tear and its location within the meniscus influenced the stresses on the tear surfaces under weight bearing. Results agree with clinical observations and suggest reasons for the inverse correlation between longitudinal tear length and healing, the inferior healing ability of medial compared with lateral menisci, and the superior healing ability of radial tears in the posterior segment of the lateral meniscus compared with other radial tears. This study has shown that meniscal tear location in addition to type likely plays a crucial role in dictating the success of non-operative treatment of the menisci. This may be used in decision making regarding conservative or surgical management.

## Introduction

Meniscal tears are the most common intra-articular injury to the knee and may occur as part of injurious events that involve rupture of the medial collateral and anterior cruciate ligaments [18]. Medial meniscal tears are observed approximately twice as frequently as lateral meniscal tears [6]. The mechanism of developing a tear in isolation from other trauma is not known; it has been, however, noted to occur during the screw-home mechanism (0°–30° flexion) and ascent from a squatting position (120°–60° flexion) [18].

As the role of the intact meniscus [15] and the resultant degenerative changes following excision have become understood, treatment for meniscal tears has evolved from meniscectomy [23] to the preservation of meniscal tissue via surgical repair [1, 2, 7, 20, 26]. Interest has also shifted to more conservative, non-operative approaches that rely on the ability of the meniscus to heal without intervention [4, 17, 28]. Stability of the ligamentous structures of the knee, tear pattern, length, location, and tear stability are parameters which may influence suitability for non-operative treatment [4, 17]. The definition, however, of tear stability varies. Stable, vertical longitudinal tears have been defined as those less than 10 mm and/or when the central portion cannot be displaced more than 3 mm [4, 17]. Similarly, stable radial tears have been defined as those shorter than the width of the inner third of the meniscus [28].

Several clinical studies have identified a subset of tears which are likely to be amenable to non-operative treatment [9, 24, 28]; success, however, in healing has been attributed primarily to local vascularity. It is probable that the biomechanics of the menisci also play a role, as the resultant stresses in the tissue surrounding a tear may result in the surfaces being compressed together or pulled apart. When the stresses result in the surfaces being compressed together, the result is likely more favourable for healing. Conversely, when the stresses result in the surfaces being pulled apart, there is likely to be propagation of the tear. The supposition is that the type of meniscal tear and its location within the meniscus will impact the potential for healing.

This is difficult to study in vivo or in vitro. In one cadaveric study, the opposing sides of longitudinal tears in the red–white zone of both lateral and medial menisci were found to remain pressed together in knee flexion [22], which lends weight to evidence that such tears are more amenable to healing on their own or with the assistance of surgical intervention.

An alternative approach is to carry out a computational study, but so far meniscal tears have only been investigated at full extension, with a limited selection of radial tears [19, 21]. None have investigated meniscal biomechanics across knee-joint flexion. Therefore, the aims of this study were to validate a finite-element model of the knee under in vivo weight-bearing conditions and use this to analyse the stress distribution through longitudinal and radial meniscal tears in three tear locations during the screw-home phase of weight bearing and utilize it to offer biomechanical explanations for observations reported in clinical studies of meniscal tears in situ. The hypothesis was that both the type of meniscal tear and its location within the meniscus would influence the stresses on the tear surfaces under weight bearing.

## Materials and methods

### Finite-element model development

Magnetic resonance images were obtained of a left knee (number of excitations = 1, echo train length = 3, slice thickness = 2 mm, slice spacing = 2 mm, matrix = 240 × 320, field of view = 140 mm with axial T1 fat saturation (fat-sat) (repetition time (TR) = 712 ms, echo time (TE) = 12 ms), coronal T1 fat-sat (TR = 730 ms, TE = 10 ms), and sagittal T1 fat-sat (TR = 796 ms, TE = 10 ms); Siemens TrioTim, Washington, DC, USA) of a single male subject (42 years; 75 kg). Thirty-five millilitres of contrast-enhancing fluid was then injected into the knee (12 ml of Isovue 300, 0.15 ml of gadolinium, 18 ml of normal saline and 4.85 ml of 0.5% ropivacaine). Following 2–3 min of unloaded knee flexion and extension, weight-bearing low-dose cone beam computed tomography (SCT) images (0.30 mm isotropic voxel size; 20 × 35 × 35 cm field of view; CurveBeam, Warrington, PA, USA) were taken to provide kinematic data for approximately 0°, 20° and 30° of knee-joint flexion. The participant was positioned with the tips of the great toes, patellae, and the anterior superior iliac spines coplanar to each other and the feet were 10° externally rotated. The work was carried out in accordance with the World Medical Association Declaration of Helsinki; however, as it was not a systematic investigation, it was not classified as research on human subjects and, therefore, did not require Institutional Research Board approval.

**Fig. 1:**
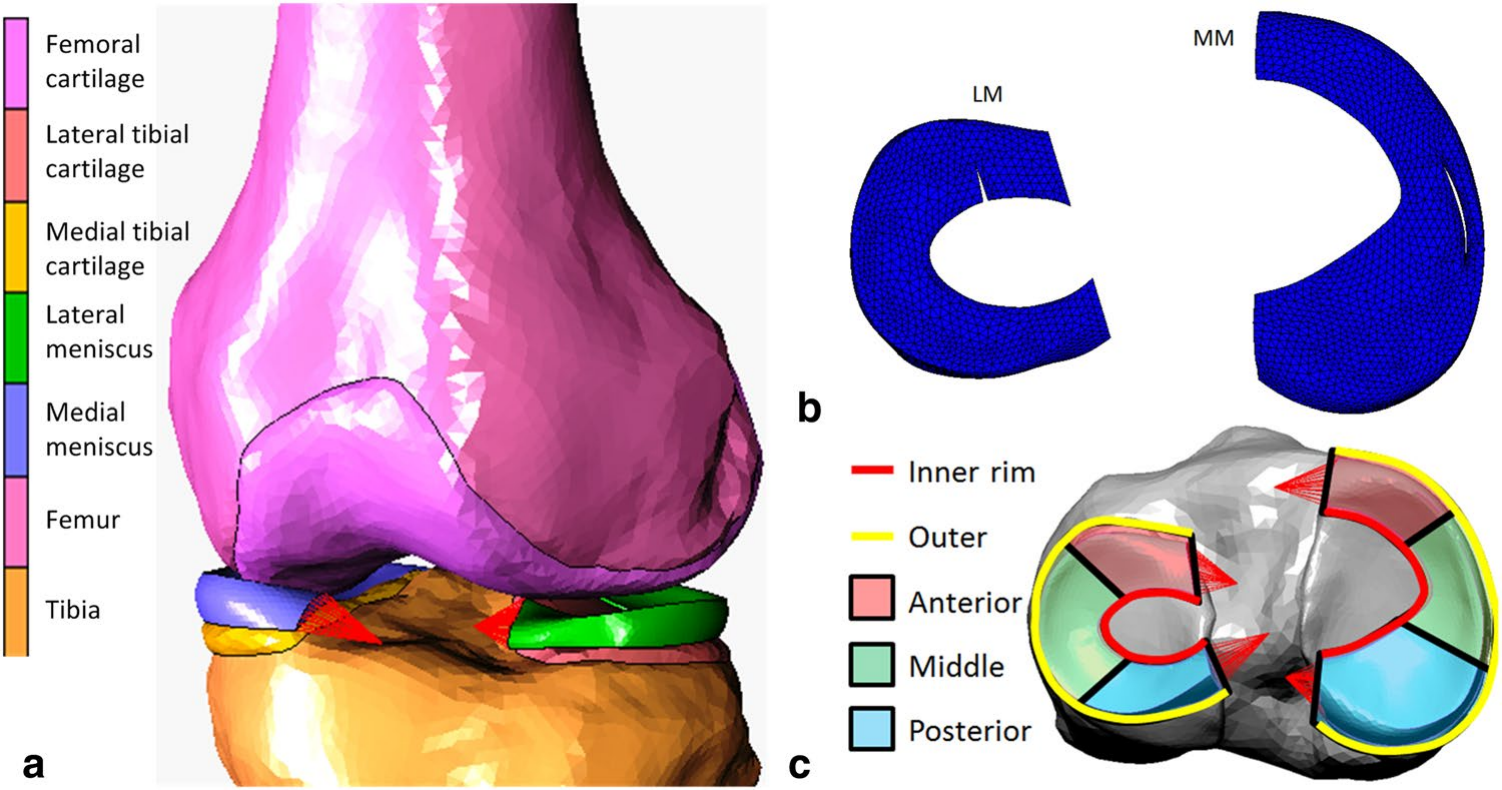
**a** Finite-element model of the tibiofemoral joint. The red lines represent the meniscal insertional ligaments. **b** Example of an unstable radial tear at the anterior region of the lateral meniscus and an unstable full-thickness vertical longitudinal tear at the middle region of the medial meniscus. **c** Anterior, middle, and posterior regions of the menisci

Images were imported into MIMICS (v. 17.0, Materialise, Leuven, Belgium) for segmentation. The femur, tibia, femoral cartilage, medial and lateral tibial cartilages, and medial (MM) and lateral (LM) menisci were segmented in the sagittal plane to capture the anteroposterior curvature of the articulating surfaces (Fig. 1a). Smoothing and Boolean subtractions between intersecting geometries were applied in 3-Matic (v. 9.0, Materialise, Leuven, Belgium); the inferior surfaces of the menisci were assumed to be congruent with the tibial articular surface. The menisci were imported into Solidworks (v. 2015, Dassault Systems, Waltham, MA, USA), where the anterior and posterior segments were terminated with flat surfaces to facilitate attachment of the insertional ligaments. The medial and lateral epicondyles and the adductor tubercle were identified on the segmented femur and correspondingly on the SCT images. Custom-written code (Matrix Laboratory, MATLAB, The Mathworks Inc., Natick, MA, USA) was used to calculate the transformations required to position the bones correctly so that they matched the kinematics of the weight-bearing SCT scans.

Geometries were meshed with linear tetrahedral elements in Mentat (v. 2013, MSC Software Corporation, Newport Beach, CA, USA). Contact between cartilage surfaces and between cartilage and meniscal surfaces allowed tangential slip with a friction coefficient of 0.02 [10]. No tangential motions were permitted between cartilage and bone interfaces. Meniscal insertional ligaments were modelled as linear non-compressive springs with insertion sites determined from the MR images. Stiffnesses for the lateral anterior, lateral posterior, medial anterior, and medial posterior ligaments were 216, 130, 169, and 207 N/mm, respectively [11]. Articular cartilage was assumed to be linearly elastic and isotropic with a Young’s modulus of 13 MPa [25] and a Poisson’s ratio of 0.42 [14]. The menisci were modelled as linearly elastic and transversely isotropic with Young’s moduli, Poisson’s ratios and shear moduli of 20 MPa, 0.2 and 8.3 MPa, respectively, in-plane, and 150 MPa, 0.3 and 57.7 MPa circumferentially [13]. Bones were modelled as rigid [12]. The tibia was constrained in all six degrees of freedom. The femur was constrained in flexion–extension, internal–external rotation, and anteroposterior translation and unconstrained in medial–lateral translation and varus–valgus angulation.

### Validation of finite-element analysis and modelling intact menisci

Simulations were run in MSC.Marc (v. 2013, MSC Software Corporation, CA, USA). Compressive loads of 375 N (representing approximately one-half body weight) and 750 N (representing approximately full body weight) were applied along the tibial long axis at 0°, 20° and 30° of knee-joint flexion. The results of meniscal movement from the simulations with one-half body weight were compared against those observed in the weight-bearing SCT images.

### Modelling meniscal tears

Meniscal tears (Fig. 1b) were created using 3-Matic on the finite-element mesh of the intact tissue. The menisci were divided into anterior, middle and posterior thirds circumferentially (Fig. 1c), and inner, middle and outer thirds radially (white avascular, red–white, and red vascular zones, respectively). Longitudinal tears were placed only at the center of the red zone, where they are most commonly observed [28]. Stable and unstable full-thickness longitudinal tears were modelled as 7 and 14 mm in length, respectively [4, 17]. Radial tears were located in the white zone, with a stable length of one-sixth of the rim width and an unstable length of one-half of the rim width [28]. All meniscal tear simulations were conducted with a compressive load of one times body weight. Maximum principal values of stress (max PCS) sampled from the inner to the outer rim in the anterior, middle, and posterior thirds of both MM and LM were used as an indicator of hoop stress. As the dominant load transmission in the articular cartilage is through compression, peak minimum principal stress is reported.

## Results

### Validation of the finite-element analysis

From 0° to 30° flexion, the LM was more mobile, moving posteriorly throughout the flexion range, whereas the MM had negligible movement from 0° to 20° flexion and moved posteriorly from 20° to 30° flexion (Table 1). Radial displacement was greater for the LM (1.8 mm between 0° and 30°) compared to that of the MM, which was negligible. Comparison of the anteroposterior displacements of the anterior and posterior meniscal segments and mediolateral displacements of the most external points of the meniscal midsection calculated by the model, with the displacements measured on the SCT images, yielded the differences, as shown in Table 2.

**Table 1:** Anteroposterior displacements of the meniscal anterior and posterior segments quantified from the model

Flexion range	Medial meniscus		Lateral meniscus	
	Anterior segment (mm)	Posterior segment (mm)	Anterior segment (mm)	Posterior segment (mm)
From 0° to 20°	0.22	0.32	− 5.9	− 3.5
From 20° to 30°	− 1.7	− 2.0	− 1.0	− 1.1

Positive values are anterior

**Table 2:** Differences in the anteroposterior displacements of the meniscal anterior and posterior segments and the mediolateral displacements of the meniscal midsection quantified from the model and spiral computed tomography

Flexion range (°)	Medial meniscus			Lateral meniscus		
	Anterior segment (mm)	Posterior segment (mm)	Midsection (mm)	Anterior segment (mm)	Posterior segment (mm)	Midsection (mm)
0–20	0.22	0.32	0.13	− 0.52	− 0.28	− 2.9
20–30	− 0.31	− 0.27	0.15	− 0.17	− 0.32	− 0.05

Positive values are anterior and medial

### Intact menisci

**Fig. 2:**
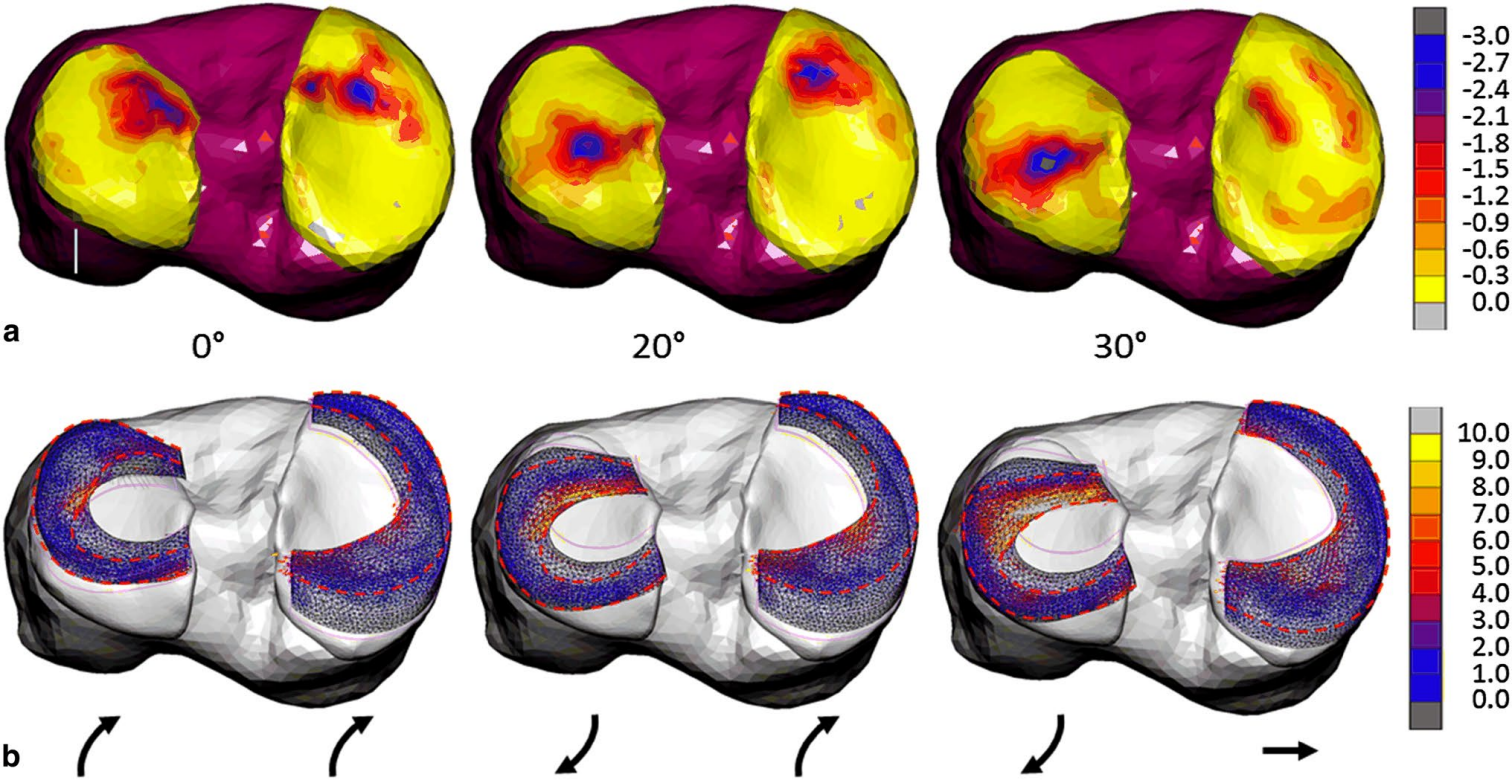
**a** Contour map of minimum principal value of stress (MPa) for tibial cartilage at 0°, 20°, and 30° flexion. **b** Tensor plots of maximum principal value of stress (MPa) in the menisci at 0°, 20°, and 30° flexion. The purple outlines show the unloaded positions of the menisci. The arrows show the direction of displacement from the unloaded positions. The red dashed outlines show approximately the region where hoop stress is dominant

From 0° to 30° flexion, the contact area between menisci and articular cartilage increased. Articular cartilage contact stress was highest on the medial side (2.7 MPa) at 0° flexion, evenly shared between medial and lateral sides (3.0 MPa) at 20° flexion, and highest on the lateral side (3.5 MPa) at 30° flexion. The posterior shift in articular cartilage contact area was larger for the lateral side (Fig. 2a).

**Fig. 3:**
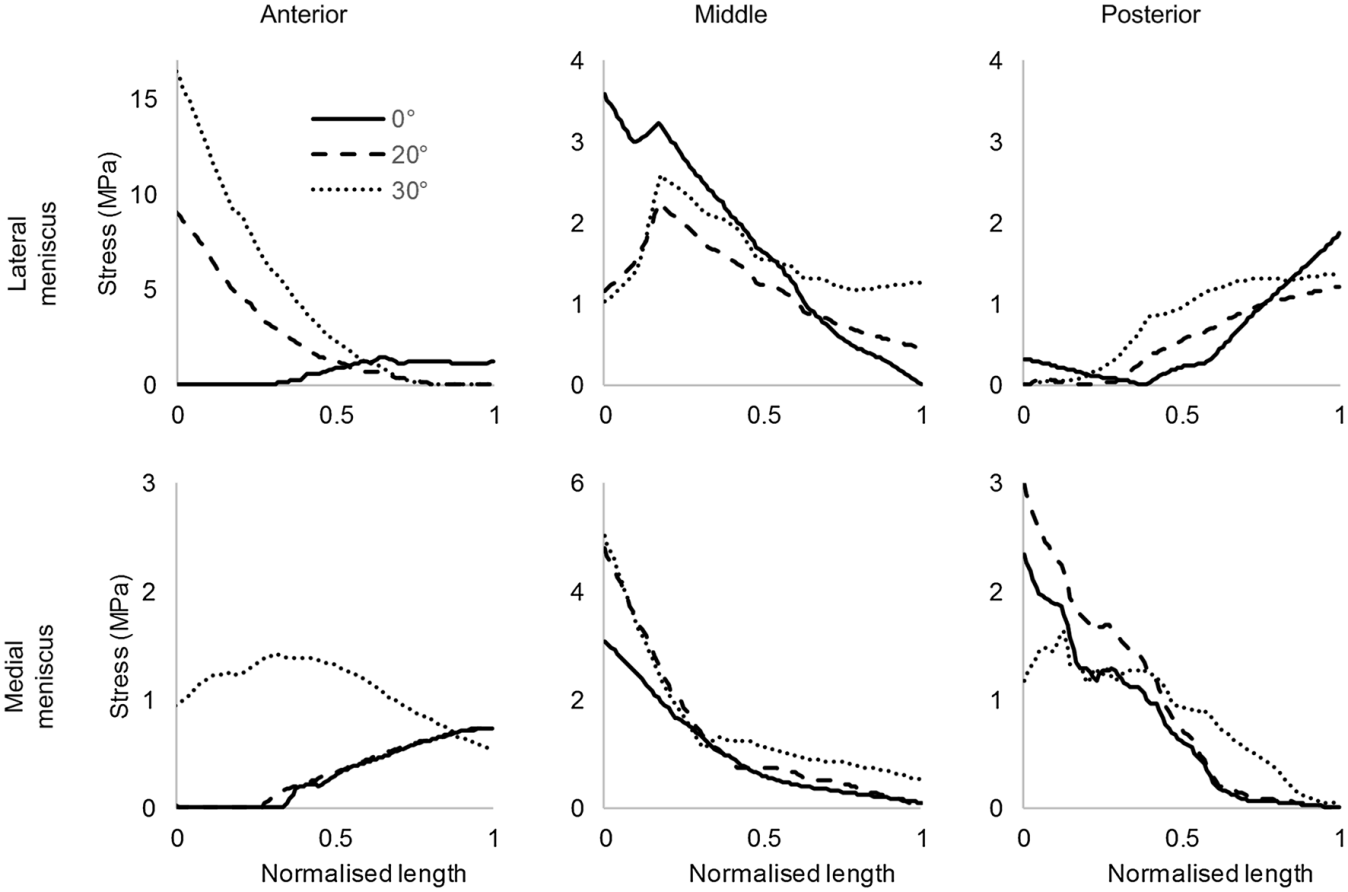
Meniscal hoop stress, measured as maximum principal value of stress, is sampled from the inner (normalised length = 0) to the outer (normalised length = 1) rim at the anterior, middle and posterior regions of the lateral and medial menisci

Deformations in the menisci from the unloaded to loaded and flexed configurations resulted in non-uniform distributions of hoop stress within the tissue. The deformations, and in turn the hoop stress distributions, were qualitatively similar at 0° and 20° flexion for the MM and at 20° and 30° flexion for the LM (Fig. 2b). In the anterior segment of the LM, the hoop stress was greatest at the outer rim at 0° flexion and at the inner rim at 20° and 30° flexion, whereas in the posterior segment it remained greatest at the outer rim for all flexion angles (Fig. 3a). Hoop stress in the midsection of the LM was predominant at the inner rim only at 0° flexion and otherwise peaked at 18% from the inner rim. Similarly, in the anterior segment of the MM, hoop stress dominated the outer rim at 0° flexion and moved towards the inner rim during flexion. In contrast with the LM, hoop stress in the midsection and posterior segment of the MM was greatest at the inner rim throughout the flexion range (Fig. 3b).

### Longitudinal tears

**Fig. 4:**
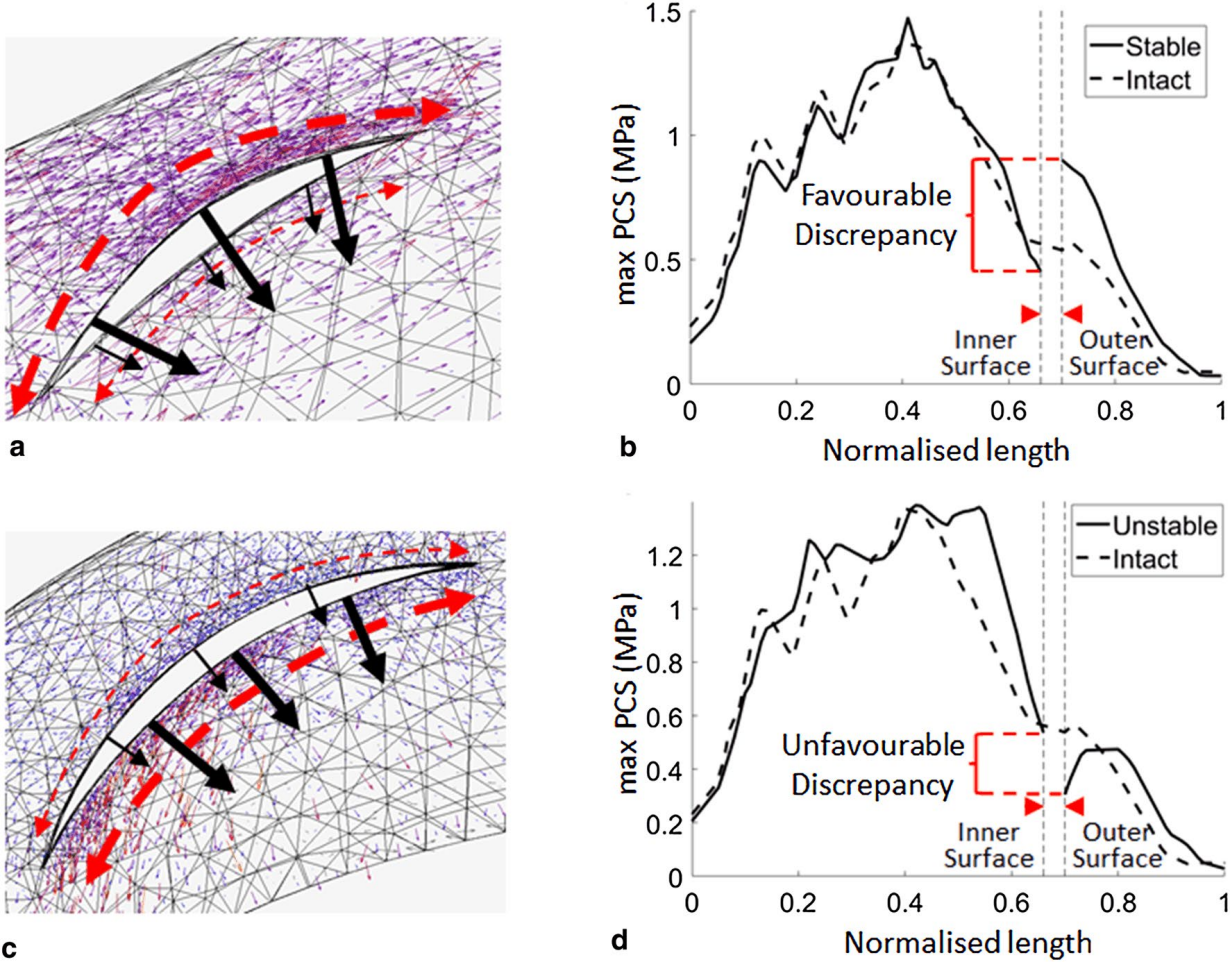
Representative (**a, c**) tensor plots and (**b, d**) maximum principal value of stress (max PCS) sampled from the inner (normalised length = 0) to the outer (normalised length = 1) rim for longitudinal (**a, b**) stable and (**c, d**) unstable tears in the posterior segment of the medial meniscus at 30° of knee flexion. Dashed arrows represent hoop stress. Solid arrows represent the component of the stress tensor acting radially inwards. Thicker arrows represent higher magnitudes

**Fig. 5:**
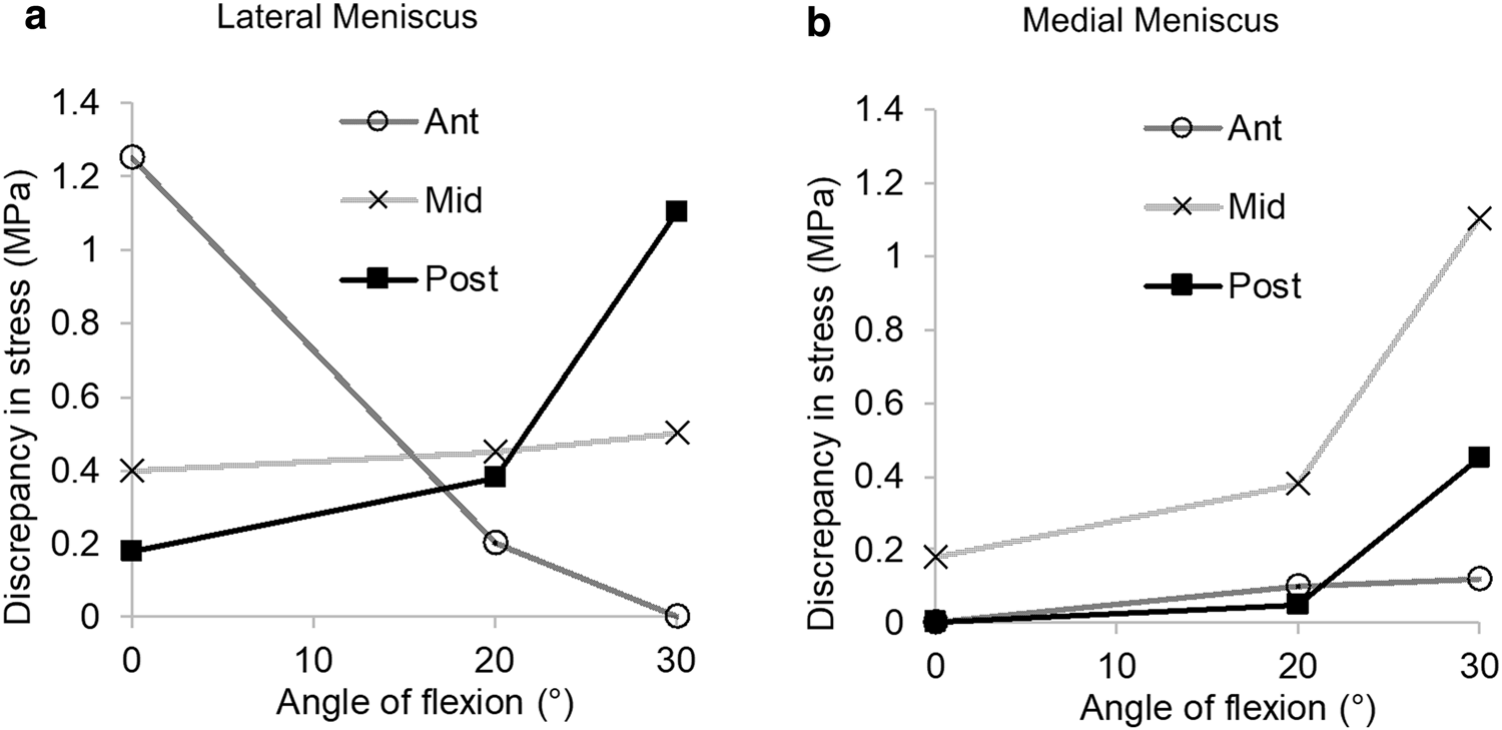
Differences in hoop stress, represented by the maximum principal stress, between the surfaces of a 7 mm longitudinal tear in all regions of both menisci plotted against flexion angle

The introduction of longitudinal tears for the most part did not alter the hoop stresses across the width of the menisci from those of the intact case. However, hoop stresses adjacent to a tear tended to be redirected to run parallel to the tear surfaces. This redirection was not equal on the two sides of the tear, resulting in a difference in stress across the tear interface. When stress at the outer surface was greater than the inner surface, the difference was deemed favourable, as the tear surfaces would be compressed together (Fig. 4a). When the stress on the inner surface was higher, the result was deemed unfavourable, as the surfaces were pulled apart (Fig. 4b). Shorter tears had more favourable differences than longer tears. Favourable differences were higher in the LM and fluctuated least in the midsections of the menisci (Fig. 5).

### Radial tears

**Fig. 6:**
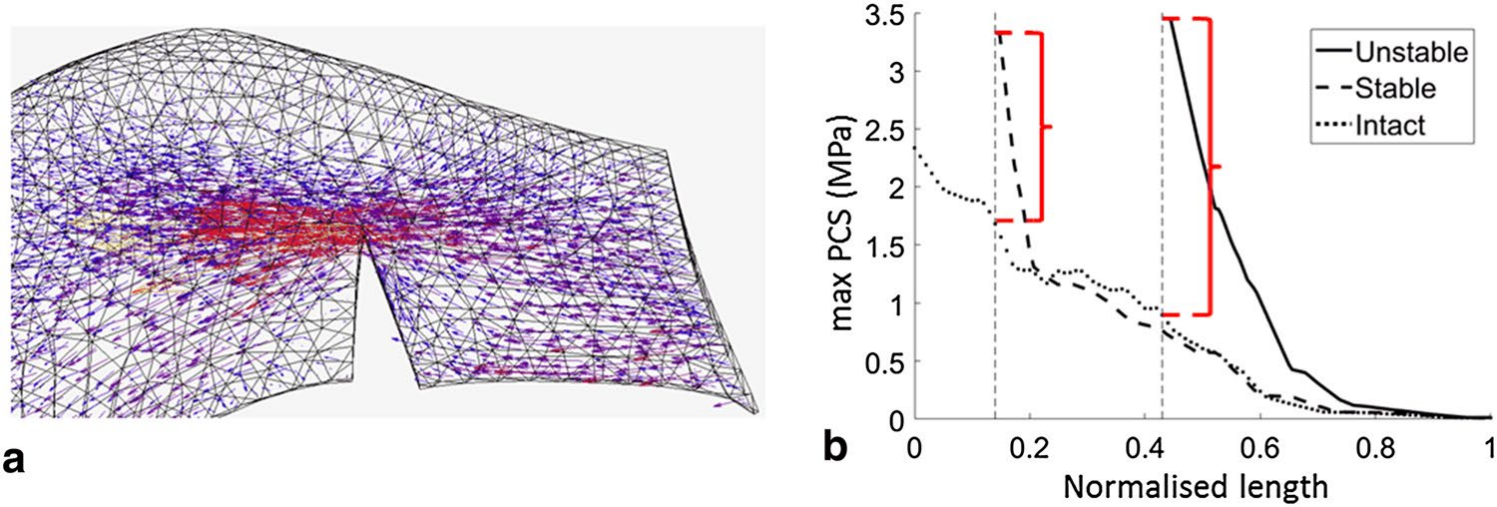
Representative **a** tensor plot for an unstable radial tear and **b** maximum principal value of stress (max PCS) sampled from the inner (normalised length = 0) to the outer (normalised length = 1) rim for stable and unstable tears in the posterior segment of the medial meniscus for 0° knee flexion. Differences between the high stresses at the tear apex and the intact condition are indicated by the red brackets

**Fig. 7:**
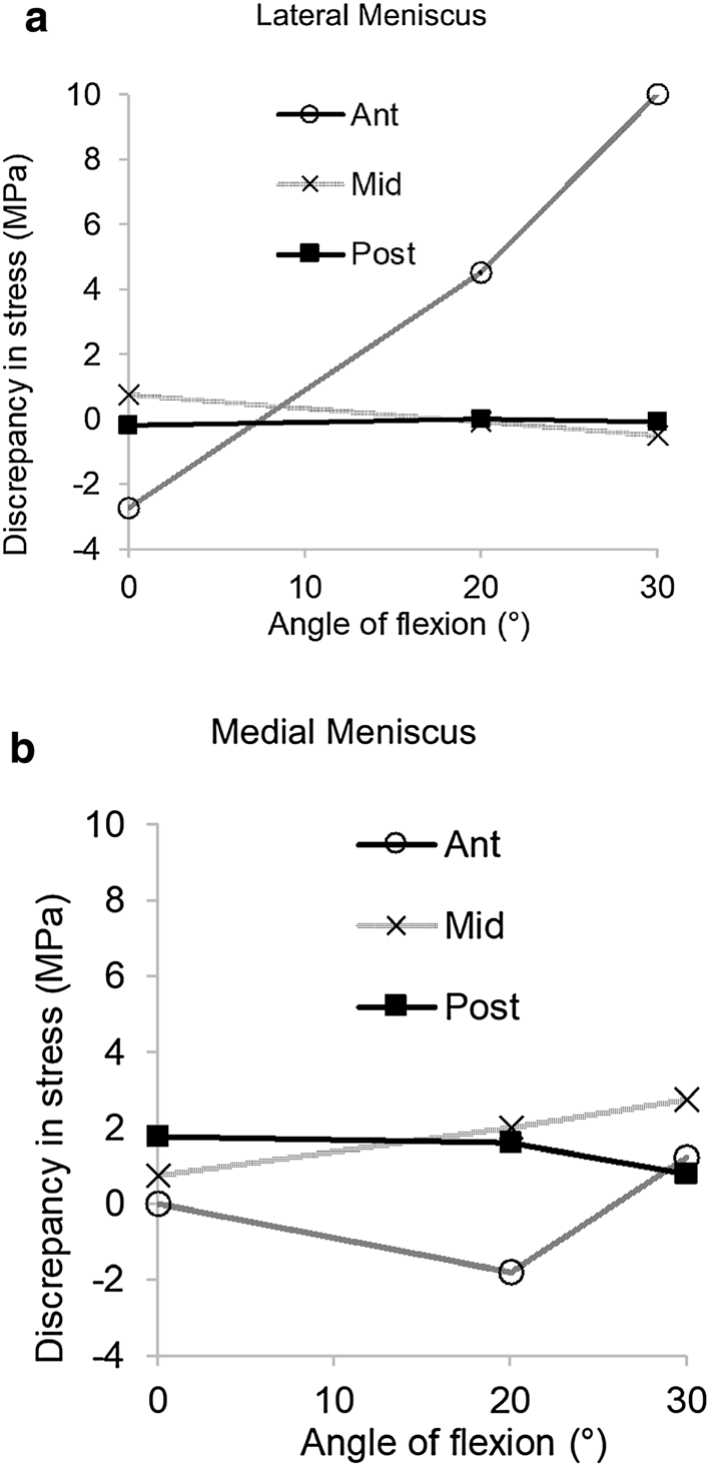
Difference in maximum principal stress at the apex of a stable radial tear in all regions of both menisci plotted against flexion angle. Positive values indicate tension and negative values indicate compression

The introduction of radial tears resulted in a significant disruption of the local stress field, including clear increases in stress at the apexes of the tears (Fig. 6a). Longer, unstable tears caused higher concentrations of stress (Fig. 6b). In the anterior segment of both menisci stresses fluctuated with knee-flexion angle between tension and compression (Fig. 7). The apexes of radial tears in the midsection of the LM experienced minimal tension or compression, while those in the midsection and posterior segment of the MM were in tension. In the posterior segment of the LM a consistent, small compression was observed.

## Discussion

Through a numerical investigation into meniscal biomechanics for intact and torn menisci for 0°, 20° and 30° of knee-joint flexion, this study has shown that both the type of meniscal tear and its location within the meniscus influence the stresses on the tear surfaces under weight bearing. The low mobility of the MM compared to the LM from 0° to 20° indicates that the model captured the screw-home mechanism that occurs during knee flexion. Between 20° and 30°, both the SCT images and the model showed a difference in posterior movements of the anterior and posterior segments of the MM, indicating that the inter-segmental distance increased. This contradicts observations by other authors working with volunteers in this flexion range [8, 16]. The LM was more mobile than the MM; posterior movement of the anterior segment of the LM was approximately six times greater between 0° and 20° than between 20° and 30°, while it was approximately three times greater for the posterior segment. In accordance with findings by others, this suggests that the anterior segment is more mobile and that the inter-segmental distance decreases with knee flexion [8, 27].

Quantitative measurements of meniscal displacements vary greatly within the literature. Studies of cadaveric knees use loading methods which may not replicate in vivo loading conditions in the tibiofemoral joint. Those which studied human volunteers examined a range of different weight-bearing positions and either do not report displacements, or do not report displacement at the knee-joint angle that we examined [5, 8, 27]. This has been acknowledged previously and the trend of comparing qualitative data between studies continues for these reasons. Therefore, we have not tested our model for validation against data from the literature, but rather from our own dataset of in vivo images.

Hoop stress was not uniformly distributed in the menisci and, as expected, its distribution changed with flexion angle. Longitudinal tears occur parallel to the circumferential collagen fibres that transfer hoop stress and only minimally disrupted the stress field. Differences in stress were observed, however, between the tear surfaces. A favourable difference, or one in which the tear surfaces were compressed together, also observed in vitro [22], results in a stable tear and possibly manifests more conducive healing conditions. This indicates that, alongside vascularity, biomechanics may play a key role in the high incidence of healing observed by Weiss et al. for non-operative treatment of longitudinal tears [28]. An inverse correlation between longitudinal tear length and meniscal healing capabilities has likewise been reported in clinical studies [2, 7]. Furthermore, simulation results showed that the MM generally has less favourable differences, suggesting it is less amenable to non-operative treatment. This may explain why a higher incidence of repair failure for longitudinal tears in the MM was observed by Barett et al. in vivo [2]. Stable tears in the midsections of the LM and MM showed the most consistent differences in hoop stress across tear surfaces suggesting that they may be most amenable to non-operative treatment. Differences in the stresses across the tear surfaces were greater for the LM than the MM, suggesting that stable tears in the LM are more likely to remain compressed throughout the screw-home mechanism.

Radial tears disrupt the circumferential collagen fibres and reduce the meniscal cross section through which hoop stress is transferred. In the model this resulted in increased stresses at the tear apex with stable tears experiencing lower stresses. Longer, unstable tears disrupt more circumferential fibres and hoop stress transmission, causing an increase in stress, and thereby increasing the likelihood of tear progression. Stresses in the MM were predominantly in tension, suggesting it is less amenable to healing than the LM. Only the posterior segment of the LM was under compression throughout the flexion range. Apart from vascularity, this may explain why radial tears in this region are most amenable to healing [4].

Limitations of this study include the idealised linearly elastic material models assigned to the menisci and articular cartilage. Nonlinear models would likely approximate better the behaviour of these structures; however, it has been shown that linearly elastic, isotropic material properties are able to model accurately bulk behaviour in cartilage [3]. In addition, it is acknowledged that healing potential is affected by factors other than biomechanics, such as biochemical processes, vascular supply, concurrent pathology, and physical activity.

Major knee ligaments were not included in the model, as the focus was on the mechanics of the menisci at specific angles of flexion. The passive and active stabilisers of the joint are responsible for guiding articular contact through motion, but they do not contribute to transfer of loads in compression and, therefore, do not affect the stress distribution over the menisci and articular cartilage at specific joint positions, such as the ones modelled here. Moreover, the model was driven based on true measures of kinematics, which capture the function of the major ligaments. The deep medial collateral and the meniscofemoral ligaments may have influenced the position of the menisci; however, these were also not included.

This study utilized patient-specific geometry and loading conditions in vivo of the same individual to develop a trustworthy finite-element model of the individual’s menisci up to 30° of flexion and used it to investigate the local mechanical environment of radial and longitudinal tears at different locations on the menisci. A strength of this study is the ability to compare directly model results with in vivo loading data of behaviour of the intact joint and menisci. A limitation is that only one patient was used, and therefore, one model was developed and analysed.

Future work should address this by imaging a number of volunteers under load and developing FE models of their tibiofemoral joints to study intact and torn meniscal mechanics. In addition, larger flexion angles should be studied, because tears have also been observed to occur at larger flexion angles, such as when ascending from the squatting position.

The results offer insight from a biomechanical standpoint into the effect of tear location and type on their healing capabilities. This has provided intuitive explanations for observations made in clinical studies and offers the potential for the stratification of treatment of meniscal tears based on imaging, enabling decisions about which tears are most amenable to conservative, non-operative treatment, which need to be surgically repaired, and which are unlikely to be reparable.

## Conclusions

In conclusion, the effect of radial and longitudinal tears on meniscal stress distribution was studied numerically. Both the type of meniscal tear and its location within the meniscus were shown to influence the stresses on the tear surface. The numerical investigation performed here is the first of its kind in studying the behaviour of stress distributions within the menisci across a range of knee-joint flexion.
